# A Case of Fibroblast Growth Factor Receptor Fusion-Positive Intrahepatic Cholangiocarcinoma With Humoral Hypercalcemia of Malignancy

**DOI:** 10.7759/cureus.58741

**Published:** 2024-04-22

**Authors:** Aditya Chauhan, Pornlada Likasitwatanakul, Ammar Ahmed, Shalamar D Sibley

**Affiliations:** 1 Department of Medicine, Division of Endocrinology, Diabetes and Metabolism, University of Minnesota School of Medicine, Minneapolis, USA; 2 Department of Medicine, Division of Internal Medicine, University of Minnesota School of Medicine, Minneapolis, USA; 3 Department of Endocrinology, Diabetes and Metabolism, Minneapolis Veterans Affairs Health Care System, Minneapolis, USA

**Keywords:** parathyroid hormone-related peptide, parathyroid hormone, hypercalcemia, humoral hypercalcemia of malignancy, cholangiocarcinoma

## Abstract

Humoral hypercalcemia of malignancy (HHM) comprises the majority of cases with malignancy-related hypercalcemia and is mediated by elevated parathyroid hormone-related peptide (PTHrP). HHM is rare in cholangiocarcinoma and has been reported only in a few case reports and series. We report a case of a 63-year-old male with a history of locally advanced fibroblast growth factor receptor (FGFR) fusion-positive intrahepatic cholangiocarcinoma who presented with recurrent HHM. The first episode of his hypercalcemia occurred 15 months after the initial diagnosis of cholangiocarcinoma and coincided with disease progression. The hypercalcemia was treated with zoledronic acid, and an FGFR inhibitor was started for the treatment of his malignancy. The second hypercalcemia episode occurred nine months later, with evidence of further disease progression. HHM is associated with poor clinical outcomes; a high index of suspicion should be present to identify and treat this complication in cases of cholangiocarcinoma promptly. With an increased understanding of the molecular alterations underlying cholangiocarcinoma, it will also be necessary to further evaluate its co-occurrence with HHM as the specific molecular alterations in this setting could lay the groundwork for targeted therapies and improve risk stratification for these patients.

## Introduction

Cholangiocarcinoma is a malignancy arising from the epithelial cells of the biliary tract and is considered to be the second most common primary hepatic malignancy. Cholangiocarcinoma in general carries a dismal prognosis [1]. Humoral hypercalcemia of malignancy (HHM) can be mediated by increased parathyroid hormone-related peptide (PTHrP) and confers a poorer prognosis. HHM in cholangiocarcinoma is very rare and has been reported only in a few case reports and series. We present a case of a 63-year-old male with fibroblast growth factor receptor (FGFR) fusion-positive metastatic intrahepatic cholangiocarcinoma presenting with recurrent HHM.

## Case presentation

A 63-year-old male, with a history of locally advanced cholangiocarcinoma, was referred from the oncology clinic to the hospital after laboratory findings were notable for mild hypercalcemia, with a corrected total serum calcium of 11.3 mg/dL (normal range:8.4-10.2 mg/dL), and hyperbilirubinemia, with a total bilirubin of 4.1 mg/dL (normal range: 0.2-1.2 mg/dL). The patient reported dry mouth, intermittent constipation, mild numbness, and tingling sensation in the hands and feet for several months. He was alert and oriented during the physical examination. The patient was a lifelong nonsmoker. There was no prior history of kidney stones or fragility fractures. There was no family history of any malignancy or calcium disorders. Medications included apixaban, metoprolol, furosemide, omeprazole, prochlorperazine, gabapentin, and magnesium oxide. Notably, the patient had completed targeted therapy with pemigatinib (FGFR inhibitor) for about two weeks at the time of presentation.

Prior oncologic history

**Figure 1:**
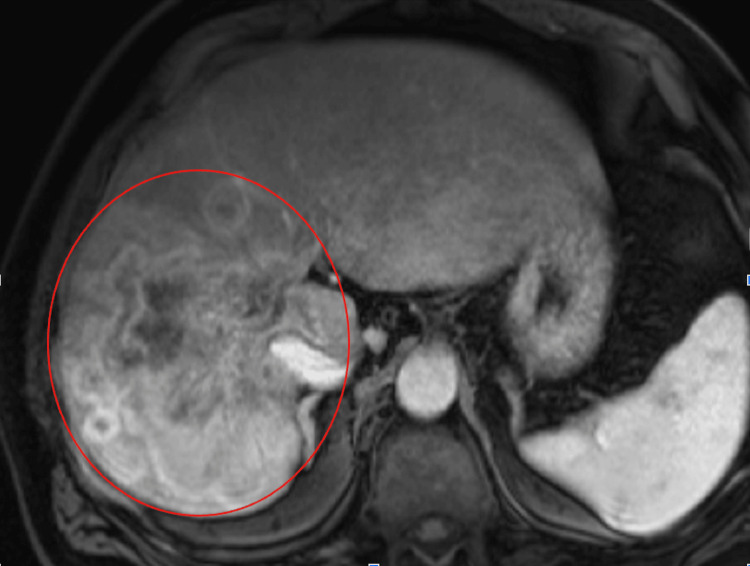
Axial view of the MRI abdomen demonstrating a large mass in the central aspect of the right lobe of the liver measuring approximately 12.0 x 9.7 x 8.6 cm extending into the caudate lobe and abutting the inferior vena cava (IVC)

**Figure 2:**
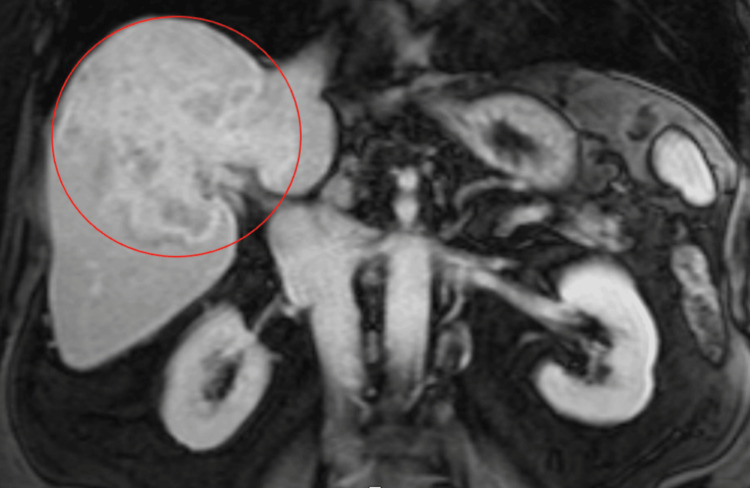
Coronal view of the MRI abdomen demonstrating a large mass in the central aspect of the right lobe of the liver measuring approximately 12.0 x 9.7 x 8.6 cm extending into the caudate lobe and abutting the IVC

Cholangiocarcinoma had been diagnosed two years earlier. The patient had initially presented with malaise, nausea, and anorexia. Magnetic resonance imaging (MRI) of the abdomen demonstrated a large mass measuring 12 cm involving the right lobe of the liver with multiple satellite lesions in the liver (Figures 1, 2).

An ultrasound-guided liver biopsy of the suspicious lesion demonstrated adenocarcinoma consistent with cholangiocarcinoma. Immunohistochemistry stains were pankeratin (+), CK7 (+), CK20 (-), arginase (-), CDX2 (-) and TTF-1/napsin (-). Foundation One CDx molecular testing from an initial tissue biopsy was pertinent for WAC-FGFR2 fusion. An initial positive emission tomography (PET) scan indicated satellite lesions in the liver and mild fludeoxyglucose (FDG) uptake at the left adrenal gland and retroperitoneal lymph nodes.

After initial diagnosis the patient received chemotherapy with cisplatin and gemcitabine for eight cycles, followed by yttrium-90 radioembolization of multifocal lesions inside the liver. Unfortunately, his disease progressed after 15 months of therapy. Around the same time, he reported having profound constipation and fatigue. Serum calcium level was 12.6 mg/dL (normal range: 8.4-10.2 mg/dL), and albumin was 2.6 mg/dL (normal range: 3.5-5.2 mg/dL) with corrected calcium levels of 13.7 mg/dL (normal range: 8.4-10.2 mg/dL). He was treated with an infusion of zoledronic acid (4 mg); at the two-week follow-up, he reported feeling great, and his corrected calcium level was down to 9.6 mg/dL (normal range: 8.4-10.2 mg/dL). The patient was subsequently transitioned to an FGFR inhibitor (pemigatinib), which controlled his disease for eight months before a progression of the disease one month prior to the current admission. His serum calcium level also remained within normal limits for the same period.

Diagnostic assessment and treatment during the current admission

Laboratory investigations are summarized in Table 1.

**Table 1:** Laboratory investigations during current admission PTH: Parathyroid hormone; PTHrP: Parathyroid hormone related-peptide; TSH: Thyroid stimulating hormone

Tests	Results	Normal range
Corrected calcium (mg/dL)	11.5	8.4-10.2
Albumin (mg/dL)	2.5	3.5-5.2
Ionized calcium (mg/dL)	6.1	4.6-5.3
Phosphorus (mg/dL)	1.6	2.3-4.7
Creatinine (mg/dL)	0.8	0.7-1.2
Alkaline phosphatase (mg/dL)	243	40-150
Total bilirubin (mg/dL)	4.1	0.2-1.2
Intact PTH (mg/dL)	7.1	8.7-77.1
PTHrP (mg/dL)	24	11-20
TSH (mg/dL)	1.47	0.35-4.94
25,OH Vitamin D (ng/mL)	19	12-50
1,25 (OH)2 Vitamin D (pg/mL)	14	18-72

The laboratory investigations were notable for a corrected total serum calcium level of 11.5 mg/dL (normal range: 8.4-10.2 mg/dL), ionized calcium of 6.1 mg/dL (normal range: 4.6-5.3 mg/dL), and phosphorus of 1.6 mg/dL (normal range: 2.3-4.7 mg/dL). The highest corrected total calcium level was 12 mg/dL (normal range: 8.4-10.2 mg/dL). The hypercalcemia workup revealed an intact PTH level of 7.1 pg/mL (normal range: 8.7-77.1 pg/mL), PTHrP of 24 pg/mL (normal range: 11-20 pg/mL), 25,OH vitamin D of 19 ng/mL (normal range: 12-50 ng/mL), and 1,25 (OH)2 vitamin D levels of 14 pg/mL (normal range: 18-72 pg/mL).

A computerized tomography (CT) scan of the abdomen and pelvis with intravenous contrast showed evidence of disease progression, with numerous hepatic lesions scattered throughout the lateral segment of the left liver lobe compared to prior scans. 

The patient was treated with IV fluid hydration and an infusion of zoledronic acid 4 mg. His serum calcium started improving and normalized within six to seven days. A review of his serum calcium over the past five years showed a close association with his cholangiocarcinoma progression, with two peaks of serum calcium requiring zoledronic acid occurring after he was found to have a disease progression. Unfortunately, the patient passed away a month later.

## Discussion

Hypercalcemia has been reported to complicate the course in up to 30% of patients with malignancy [2]. It is more commonly seen in advanced disease and is associated with a poorer prognosis [2]. Approximately 80% of malignancy-related hypercalcemia is due to HHM, which is often mediated by increased PTHrP [2]. Other proposed mechanisms include local osteolytic hypercalcemia from bone metastasis, excess extrarenal production of activated vitamin D(1,25(OH)2D), and ectopic PTH production. HHM is mostly seen in squamous cell carcinomas such as the lung, head and neck, esophagus, and cervix [3,4]. HHM in cases of cholangiocarcinoma is rare and, to our knowledge, has only been reported in a few case reports [5-9].

Cholangiocarcinoma has been anatomically classified into intrahepatic, distal, and perihilar with intrahepatic being 10-20% of all cases [10]. HHM has been reported predominantly in cases of intrahepatic cholangiocarcinoma [5]. Unresectable cholangiocarcinoma carries a dismal prognosis in general [10]. The demographic characteristics and anatomical classification of this case are in line with what has been previously reported in the literature. Literature suggests a median survival from diagnosis of hypercalcemia to be 3-5 months [5,11]. However, our patient lived for approximately 10 months after the initial diagnosis of hypercalcemia. The improved life expectancy could be due to advancements in the targeted therapies in recent years. Another point that merits discussion is that certain molecular tumor marker profiles may predispose patients to develop HHM. Konstantinidou et al. in their study subclassified the cohort on the basis of common molecular alterations associated with cholangiocarcinoma. Isocitrate dehydrogenase 1 (IDH1) that mutated intrahepatic cholangiocarcinoma was associated with HHM [11]. A few cases of HHM in patients with FGFR2 fusion (21%) have been reported, but no statistically significant associations between FGFR2 fusion-positive cholangiocarcinoma and HHM have been found [11].

PTHrP is homologous to PTH and shares almost identical amino acids allowing both peptides to activate the same PTH/PTHrP receptor, which is a G protein-coupled receptor. However, both of these ligands have distinct activation and binding sites, which make them differ slightly in their biological actions [12]. PTHrP promotes bone resorption and enhances renal absorption of calcium and excretion of phosphorus, leading to hypercalcemia and hypophosphatemia. However, unlike PTH, PTHrP does not stimulate 25,OH vitamin D activation and does not have an effect on intestinal absorption of calcium. Literature suggests that elevated PTHrP might be an indicator of aggressive disease and reflects poor prognosis, especially in patients less than 65 years of age [13]. A recent case report and literature review of HHM in cholangiocarcinoma by Ito et al. highlighted similar findings in regard to PTHrP and associated poor prognosis [5].

There should be a low threshold for suspicion of hypercalcemia in patients with cancer as symptoms such as nausea, vomiting, loss of appetite, and cognitive changes may overlap with other causes of these symptoms in this population. This may lead to delayed diagnosis and treatment of hypercalcemia. As in our case, HHM is characterized by an elevated PTHrP, low PTH, and a low or normal 1,25(OH)2D [14]. In addition to treatment of the underlying malignancy, moderate-to-severe hypercalcemia (serum calcium more than 12 mg/dL) should be treated with acute measures. The two main pillars of treatment in HHM include promoting renal calcium excretion and reducing bone resorption. Therefore, fluid replacement is a critical component of HHM management. Although the evidence is not very strong, the Endocrine Society recommends IV denosumab over bisphosphonates in this setting. Denosumab acts by inhibiting receptor activators of nuclear factor kappa beta (NFkB ligand) (RANKL), thereby inhibiting bone resorption, and the median duration of this effect is estimated to be around 104 days [15]. The recommendations are based on prospective studies performed on patients with solid tumors and evidence of bone metastasis. They note that the use of denosumab was associated with decreased incidence of hypercalcemia of malignancy, reduced risk of recurrent hypercalcemia, fewer skeletal-related adverse events, and greater suppression of bone turnover markers. However, none of the studies showed any difference in overall survival. Calcitonin should be used as an adjunctive therapy in patients with calcium above 14 mg/dL [15]. In our case, after the first zoledronate dose, there was no recurrence of hypercalcemia for around nine months. The recurrence of hypercalcemia coincided with disease progression.

## Conclusions

HHM in cholangiocarcinoma is very rare and is associated with poor clinical outcomes. A high index of suspicion for hypercalcemia should be present when evaluating patients with cholangiocarcinoma given that the presenting symptoms can be nonspecific, thus delaying the diagnosis and treatment of HHM in this setting. With an increased understanding of molecular alterations associated with cholangiocarcinoma, it will also be important to further evaluate the specific molecular alterations that underlie the co-occurrence of cholangiocarcinoma with HHM. The application of this information could be huge, allowing for improvement in risk stratification and potentially the development of more targeted therapies to treat patients with cholangiocarcinoma-associated HHM.
